# Climate change induced human displacement in Bangladesh: Implications on the livelihood of displaced riverine island dwellers and their adaptation strategies

**DOI:** 10.3389/fpsyg.2022.964648

**Published:** 2022-10-13

**Authors:** Babul Hossain, Guoqing Shi, Chen Ajiang, Md. Nazirul Islam Sarker, Md. Salman Sohel, Zhonggen Sun, Qi Yang

**Affiliations:** ^1^Management Science and Engineering, Hohai University, Nanjing, China; ^2^National Research Center for Resettlement, Hohai University, Nanjing, China; ^3^Research Center for Environment and Society, Hohai University, Nanjing, China; ^4^School of Social Sciences, Universiti Sains Malaysia, Pulau Pinang, Malaysia; ^5^Asian Research Center, Hohai University, Nanjing, China; ^6^Department of Sociology, School of Public Administration, Hohai University, Nanjing, China

**Keywords:** climate change perception, natural disasters, livelihood vulnerability, livelihood resilience, adaptation obstacle, internally displaced char dwellers

## Abstract

In Bangladesh, many people are being displaced in riverine island (char) areas every year due to climate change and its associated natural catastrophes. This study intends to investigate the impact of climate change on internally displaced char people’s lives and livelihoods along with local adaptation strategies and hindrances to the coping mechanism. Data have been collected from 280 internally displaced households in two sub-districts. A mixed-method approach has been considered combined with qualitative and quantitative methods. The results disclose that frequent flooding, riverbank erosion, and crop loss are the leading causes for relocation, and social relations are impeded in the new place of residence. Increasing summer and winter temperatures, recurrent flooding, severity of riverbank erosion, and expanding disease outbreaks are also important indicators of climate change identified by displaced people, which are consistent with observed data. This study also reveals that almost all households come across severe livelihood issues like food shortage, unemployment and income loss, and housing and sanitation problems due to the changing climate associated with disasters in the former and present places. In response to this, the displaced people acclimatize applying numerous adaptation strategies in order to boost the livelihood resilience against climate change. However, fragile housing, financial conditions, and lack of own land are still the highest impediments to the sustainability of adaptation. Therefore, along with the government, several organizations should implement a dynamic resettlement project through appropriate scrutiny to eradicate the livelihood complications of internally displaced people.

## Introduction

Climate change has confronted the world with huge complications and threats in the 21st century (Tajrin and Hossain, 2017). Developing countries are not exception and increasingly vulnerable due to the enhancing regularity and intensity of natural hazards (IPCC, 2014; Islam et al., 2019; Yousafzai et al., 2022). The shifting trend of temperature, rainfall, sea level rise, and the alteration of severe climate indicators are all manifestations of climate change and its consequences (Chen and Mueller, 2018; Hossain et al., 2020b). Climate change has a widespread impact on world economic, social, and political activities and disrupts the people’s way of life massively (Brooks et al., 2009; Du et al., 2013; Bergquist et al., 2019). As a result, one of the myriad problems that people face worldwide is the harmful effects of climate change (Wang et al., 2019; Uddin et al., 2022). It has already been documented that climate change, which has a detrimental consequence on the lives and livelihoods of the world population, is predicted to be one of the most significant risks to economic progress of developing countries (IPCC, 2014; Tol, 2018; Wrathall et al., 2019). Natural hazards increase the number of displaced people, and climate change hastens these situations (Ahmed, 2018; Wang et al., 2020). According to Brzoska and Fröhlich (2016), the amount of environmental or climate change-induced displaced individuals is higher than political and war refugees. Furthermore, according to the IPCC (2019), climate change-related impacts could lead 150 million people to be displaced by 2050.

Bangladesh is one of the countries that are most vulnerable and imperiled to climate change due to its distinctive geographical position, brittle socioeconomic settings, expanding populace, high poverty, and low degree of financial and technological capabilities (Shahid and Behrawan, 2008). In Bangladesh, the average temperature is rising day by day, and by 2030 and 2050, the temperature will have increased by 1.0 and 1.4°C, respectively, owing to climate change (IPCC, 2014; Roth et al., 2021). In terms of extreme weather, it is rated as the fifth most susceptible state in the world. As a result, approximately every year, more or less, this country is subjected to repeated severe climate phenomena, such as floods, riverbank erosion, cyclones, saline intrusion, landslides, storm surges, and droughts (Rakib et al., 2018). It exacerbates abundant problems, including severe direct and indirect life and livelihood issues (Kabir R. et al., 2016). However, one of the most catastrophic implications of climate change is that people are being forced to flee their homes, lands, and livelihoods due to natural disasters caused by climate change (Barua et al., 2017; Islam et al., 2022). Many tens of millions of people will be displaced due to these processes in the coming years (Rahman, 2021). Bangladesh is very susceptible to the effects of climate change, which might force up to 30 million people to flee their homes by 2100 if sea levels rise to the projected 80 cm or higher (Barua et al., 2017). People who have been displaced or who have migrated suffer a severe lack of human rights facilities and fierce competition for unbiased access to wealth, as well as the effects of fast urbanization, water shortages, lack of power, hardship, and the rising intensity and occurrence of catastrophes (Goodwin-gill and Mcadam, 2017). Also, internal migration or displacement can lead to conflict (Malpeli et al., 2020). On the contrary, Bardsley and Hugo (2010) recommended that internal migration be worthwhile tactics as acclimatization to contend with the rising effects of climate change.

However, in Bangladesh, char (riverine island) is one of the highly susceptible locations to climate change owing to its proximity to flood-prone rivers (Hossain et al., 2021). Char land is a type of land that forms over 2–3 years due to continual riverbank erosion and sediment deposition in large rivers and shoreline zones. This land is mainly cut off from the mainland (Karim and Thiel, 2017). This region is historically known as the most ignored and underprivileged (Emdad Haque and Zaman, 1993). Regularly, this part is highly exposed to natural calamities, such as floods and riverbank erosion (Hossain et al., 2021). Furthermore, it is estimated that around 4–5% of the populace of Bangladesh lives in the char land. Most char households are directly or indirectly involved in agriculture (Hossain et al., 2019). Households living on islands are thought to be the highly exposed to the effects of climate change (Shah et al., 2013). Climate change-associated disaster is destroying their crops, farmland, and houses. Char inhabitants are more defenseless, highly poor, and food uncertain due to these distinctive characteristics. Because char dwellers have restricted access to essential requirements, including food, agriculture, education, health, and finance, they are more prone to become impoverished. Every year, the char dwellers face extreme climate change-related threats, such as floods, riverbank erosion, and drought. As a result, these climate change-related push factors induced huge displacement among the char dwellers.

Therefore, it is documented that the displaced people are confronted with massive complications regarding lives and livelihood after being displaced to a new place from their ancestral location (Elshater, 2021). The significant climate change-induced impact on various livelihood assets is rising day by day (Hossain et al., 2020a). Paudel (2010) stated that food uncertainty and malnutrition had increased massively due to the lack of work opportunities in the new places. In addition, extreme climate events have increased various common diseases, such as cold and cough, fever, headache, heart stock, mental disorders, back pain, energy loss, breathing problems, and tonsil (Haque et al., 2013). In addition, housing and sanitation issues, deterioration of social relations, and so on lead to overwhelming consequences after being displaced in a new location (Hossain et al., 2020a). For this reason, this study has been concentrated on the char dwellers displaced in a new location. A majority of these people are dwelling under ultra-poor conditions, including a low level of income and occupation. It is well known that catastrophes disproportionately affect the underprivileged (Penning-Rowsell et al., 2013), and climatic hazards are projected to wreak devastation on these individuals the most. Yet, such people do not have adequate defense strategies (Brouwer et al., 2007).

Internally displaced people (IDPs) more or less form adaptation techniques in response to the livelihood complications triggered by climate change and its associated disasters. Adaptation tactics differ from one place to another place and from one culture to another (Rahman and Hickey, 2019; Khan et al., 2020). Nevertheless, adaptation is essential for defenseless nations like Bangladesh to increase adaptive capacity and reduce societal susceptibility in the face of climate change (Ausden, 2014; Vij et al., 2018). Adaptation systems are developed based on the presence of the individuals’ technological, social, environmental, economic, and physical resources. To cope with the effects of changing climate, impoverished rural communities have heavily relied on adaptation tactics. However, without a proper understanding of climate change impacts and local adaption techniques among char dwellers, the approach may not be effective in strengthening resistance to adverse effects of climate change (Tang and Hailu, 2020). On the contrary, several types of barriers, such as cultural, social, financial, environmental, and organizational obstacles, reduce the adaptability capacity and increase (Lahsen et al., 2010). Some studies detected that the scarcity of political will and harmonization among diverse institutions, insufficiency of monetary provision, restricted possessions, and paucity of awareness have all been recognized as impediments to viable adaptation (Runhaar et al., 2012).

A number of studies on climate change-associated issues, such as health, livelihood, displacement, and resettlement, including adaptation strategy, have formerly been carried out (Zaman, 1989, 1993; Pouliotte et al., 2009; Haque et al., 2013; Alam, 2017; Fang et al., 2018; Liu et al., 2020; Hossain et al., 2021). But, a very little focus has been placed on the impacts on the livelihood of IDPs caused by climate change and its associated hazards. More precisely, in the case of Bangladesh, a specific focus on the impact of climate change on lives and livelihood and adaption practices of riverine island dwellers is still lacking. As a result, this study aims to fill some of this gap by examining the impact of climate change on lives and livelihood from displaced people’s perspectives and by finding local adaptation mechanisms in char district areas of Bangladesh, with particular attention to the weaknesses and limitations of their coping strategies. The number of internally displaced individuals is growing, so it is crucial to discover a way to mitigate the effects of significant climate change in riverine char districts areas of Bangladesh. Furthermore, it is crucial to know the impediments to adaption practices among displaced people’s communities in disaster-prone areas to facilitate proper climate change adaptation. This study will contribute to the existing knowledge of acclimatization efforts against the consequences of climate change. In addition, it will help determine the climate change barriers to displaced dwellers’ adjusting techniques in Bangladesh.

## Materials and methods

This study followed a mixed-method approach incorporating qualitative and quantitative approaches. Hence, a questionnaire survey and an interview guide were adopted for the quantitative and qualitative approach, respectively. In addition, an in-depth literature review for secondary data was carried out before collecting data.

### Study area and location

Gaibandha is one of the most natural disaster-prone districts in Bangladesh. Every year, climate-related disasters such as floods, riverbank erosion, cyclones, and other natural disasters strike this region (Islam, 2018). Furthermore, the dwellers of this area are exposed to disasters such as summer storms, floods, and riverbank erosion during the rainy season, as well as summer and spring droughts, and winter cold waves (Hossain et al., 2020a). Climate change is wreaking havoc on their crops, farming lands, and homesteads. The residents of this area are more vulnerable, highly impoverished, and food insecure as a result of these disasters. They are also more prone to fall into poverty since they have little access to essential needs. The effects of climate change have a bad influence on their incomes and work chances (Brammer, 2014). Due to this, the lives and livelihood of the people of this region are severely disrupted every year (Sarker et al., 2019). As a result, many people are internally displaced from one place to another, which has become a regular phenomenon in this region (Hossain et al., 2020a). This is why Gaibandha district has been purposively selected for this study.

**FIGURE 1:**
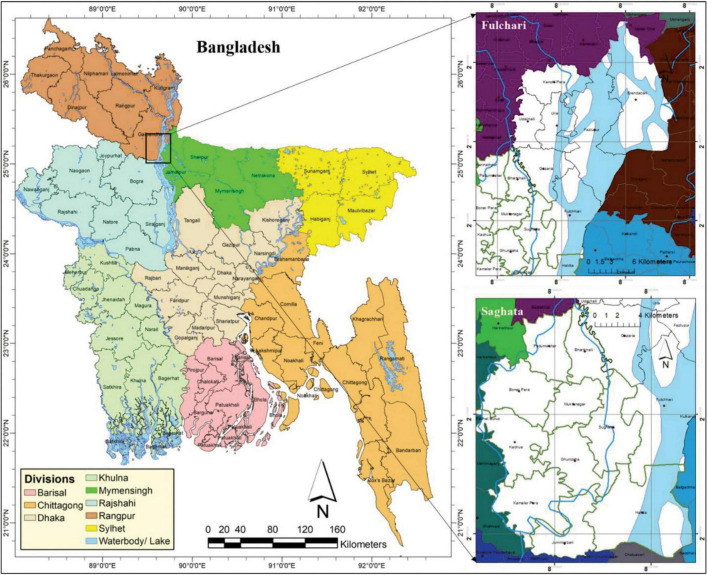
Location of the study area.

Gaibandha district is located in the Rangpur Division. It is surrounded on the north by Kurigram district and Rangpur district, on the east by Kurigram district and Jamalpur district, on the south by Bogra district, and on the west by Joypurhat district, Dinajpur district, and Rangpur district (BBS, 2011). Gaibandha district lies between 25°02′ and 25°39′ north latitudes, and between 89°11′ and 89°46′ east longitudes. The total area of the district is 2,114.77 sq. km (816.00 sq. miles) (BBS, 2011). To meet the purpose of this study, Fulchari and Saghata upazilas have been chosen purposively as the study area from the seven sub-districts (upazila) in the Gaibandha district (Figure 1) since this area is very adjacent to the Jamuna–Brahmaputra River basin zones.

### Sampling and data collection

The following statistical procedure was used to estimate a representative sample size for this investigation (Cochran, 2017):


$$n=\frac{z^{2}\times p\times q\times N}{e^{2}\;(N-1)\;+\;z^{2}\times p\times q}=280$$

Here, *n* = Sample size, N = total number of households, z = confidence level (at 95% probability = 1.96, *p* = estimated population proportion (0.5 this maximizes the sample size), *q* = 1-p, e = error limit of 5% (0.05).

Therefore, a total of 280 internally displaced people (IDP) households (140 from each sub-district) were selected by simple random sampling among the IDP communities from the two sub-districts (total 1,029 households). The field surveys were conducted using a semi-structured questionnaire, and a pilot survey was conducted to learn the concerns to be investigated in this study. Thus, two sets of open-ended and close-ended interview questionnaires (IDPs and KIIs) were used to conduct a field survey in terms of the pilot survey, field visit, literature review, and specialist view concerning the study aims.

Therefore, quantitative data were collected from 280 IDPs by using a questionnaire survey. On the other hand, qualitative data were collected from focus group discussions (FGDs), depth interviews, KIIs, and observations. A total of six FGDs (one male group and one female group from each sub-district) were carried out, where 8–12 displaced individuals take part in each focus group discussion. In some of the cases, some of the people did not agree to interview in front of the other people, for whom depth interviews were conducted to know the actual condition of the displaced people in the study area. In addition, eight key informant interviews (KIIs) were carried out with several officials from the GO and NGO sectors concerned with climate change, migration, and IDPs along with local representatives from Fulchari and Saghata. A purposive method was applied to select the key informants experienced in climate change, natural disasters, migration, and adaptation strategies. The survey was conducted from October 2018 to March 2019.

Ethical considerations were given much attention. During the fieldwork, the researchers introduced themselves and explained the reason for their visit. Furthermore, all respondents gave their consent before the interview session, recording, or taking a snapshot of their activity. The respondents were informed of the study goal and promised that their identities would not be revealed.

### Data analysis techniques

After gathering all the data through interview sessions with the respondents, the gathered data were examined in accordance with the rationale of this study. Statistical Package for the Social Sciences (SPSS) was used to analyze the quantitative data. The univariate and bivariate analyses were carried out for the displaced people’s responses in the context of climate change, migration, and acclimatization strategies, along with hindrances to adaptation in the migrated places in Bangladesh. On the other hand, the qualitative data figured out via textual and document analysis. Tables, charts, and graphs were categorized, at the same time, to make the material more pertinent and accessible to the person who reads. Furthermore, the investigators clarified in terms of the findings and observations made throughout the primary and secondary data analyses, as well as interviews with informants.

### Measurement of climate change impact and adaptation practice

The climate change impact was measured in this study through five livelihood assets, namely, human capital, social capital, financial capital, physical capital, and natural capital. In addition, several variables (Table 4) under the five livelihood capitals were also considered to comprehend the intensity of the climate change impact on the IDPs. Furthermore, climate change impacts on displaced people’s way of life were also analyzed based on the previous and present locations. In addition, five scales such as no, slightly, medium, and large have been considered to analyze the climate change and its associated hazard impacts regarding the displaced people’s lives and livelihood. On the contrary, the IDPs’ acclimatization approaches for lessening climate change impacts were considered using 20 variables. Concerning this, three scales like low, medium, and high were also taken into account based on households’ income patterns to explore the real scenarios of the displaced people (Table 5). In addition, this study found two categories of adaptation strategies, namely, (1) individual-level adaptation (ILA) and (2) planned adaptation (PA), that mainly IDPs follow to cope with the climate change impacts on livelihood (Hossain et al., 2021).

## Results

### Socioeconomic conditions of displaced people

The main purpose of this section is to understand the respondents’ socioeconomic characteristics in the study areas. This part has been designed based on the socioeconomic status of individuals influenced by climate change, which is associated to their age, gender, education, main occupation, and household income, in order to get in-depth evidence about the participants.

In this study, among the entire respondents in the study area, 50% were male and 50% were female, in which a majority of the respondents were young and middle-aged. In addition, 36.07% of the respondents were illiterate, while 38.21, 14.29, and 11.43% of the participants had primary, secondary, and above the secondary level of education status. Furthermore, the respondents were engaged in several occupations in the study area, such as farmer, day laborers, housemaid, rickshaw puller, street hawker, beggar, and bricklayer service (see Table 1). On the other hand, Table 1 also reveals the respondents’ personal monthly income profile, where the respondent’s monthly income was not good enough. Most of the respondents’ monthly income was below 8,000 BDT, and only 2.5% of the respondents’ personal income was above 8,000 BDT. On the contrary, 34.64 and 7.14% of the respondents’ monthly income were 1,000–5,000 BDT and above 10,000 BDT, respectively. But the majority (58.21%) of the monthly household income was 5,000–10,000 BDT.

**TABLE 1:** Socioeconomic characteristics of the respondents.

Characteristic	Scoring system	Categories	Respondents		Mean	SD
			F	%		
Gender	Code	Male (1)	140	50.00	1.5	0.5
		Female (2)	140	50.00		
Age	Years	Young (18–35)	113	40.36	43.59	20.32
		Middle (36–53)	127	45.35		
		Old (54–70)	40	14.29		
Education	Year of schooling	Illiterate (1)	101	36.07	4.47	5.26
		Primary level (1–5)	107	38.21		
		Secondary level (6- 10)	40	14.29		
		Above secondary (> 10)	32	11.43		
Primary occupation	Code	Housewife (1)	53	18.93	4.28	2.42
		Day labor (2)	34	12.14		
		Farmer (3)	26	9.29		
		Housemaid (4)	33	11.79		
		Rickshaw puller (5)	41	14.64		
		Street hawker (6)	27	9.64		
		Beggar (7)	35	12.5		
		Bricklayer (8)	24	8.57		
		Service (9)	7	2.5		
Personal monthly income	BDT.	≤ 2,000	103	36.79	3,376.79	2,000.00
		2,000–4,000	91	32.50		
		4,000–6,000	49	17.50		
		6,000–8,000	30	10.71		
		> 8,000	7	2.5		
Household monthly income	BDT.	1,000–5,000 (low)	97	34.64	6,298.21	2,712.31
		5,000–10,000 (Medium)	163	58.21		
		> 10,000 (High)	20	7.14		

Field survey (1 USD = 95 BDT).

### Drivers of prompting displacements from ancestral place

**FIGURE 2:**
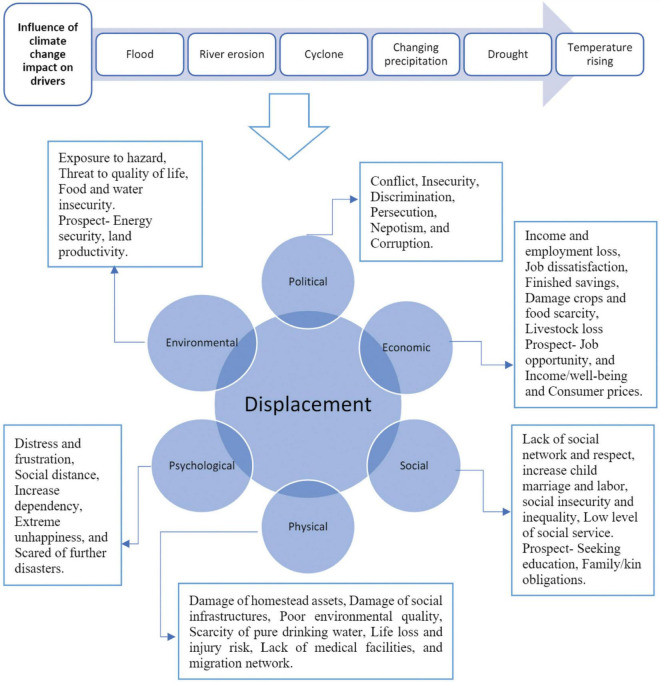
Drivers and factors of displacement from riverine island areas in Bangladesh.

This section depicts the drivers and factors of displacement of riverine island dwellers from their ancestral locations. This study revealed that six drivers, namely, environmental, social, economic, physical, psychological, and political drivers, induced displacement behavior of the char dwellers enormously (Figure 2). Climate change and its associated disasters like flood and erosion are the significant influencing drivers and factors of displacement. The FGDs unfolded that loss of agriculture, unemployment and income loss, damage of homestead, scarcity of food, freshwater crisis, problem of sanitation, and less educational opportunity were the most important causes for being relocated from their ancestral locations.

In addition, qualitative findings revealed most respondents were displaced from a riverine island located in Fulchari and Saghata upazilas of Gaibandha district. These zones are highly vulnerable to frequent flood disasters, riverbanks, and other natural calamities. Every year, many people lose their assets, including land, livestock, and way of living options, owing to the implications of several natural catastrophes, especially riverbank erosion caused damage at an enormous scale. For example, substantial loss of properties and harm to living opportunities were detected owing to flood events in 2017. A respondent stated his/her opinion in this regard as follows:

*“I and my family were decided that not to leave our ancestral homestead at any cost in the face of natural calamities’ damages because we thought that we wouldn’t confront any significant issues. But after 2 years later, we encountered an overwhelming 2017 flood disaster in our living area, and finally, we had to leave our home due to lost our homestead land caused by the river erosion. We not only faced this problem alone but also our neighbors met the same problem. Since we are a poor man, therefore, we were no bounds of sorrows in our life after being forced displacement from our home, which made us more vulnerable” (Interviewee #59).*

### Conditions of civic amenities in the prior and present locations

Table 2 demonstrates the displaced people’s opportunities and further civic amenities in former and current places. After the resettlement, the displaced people were confronted with the actualities of various social circumstances and amenities in the dwelling area.

**TABLE 2:** Opportunities and other civic amenities in prior and current locations.

Amenities	Condition	Very good	Good	Neither good nor bad	Very bad	Bad	Comment
Communication system	PvC	–	5.36	14.64	51.07	28.93	+
	PsC	19.29	67.5	10.00	–	3.21	
Sanitation amenities	PvC	–	12.14	14.29	45.00	28.57	+
	PsC	2.50	67.50	9.29	9.64	11.07	
Drinking water deliver	PvC	12.14	2.50	9.29	57.14	18.93	+
	PsC	9.64	50.36	14.64	–	25.36	
Health care services	PvC	–	–	4.64	64.29	31.07	+
	PsC	5.36	23.93	33.21	2.5	35.00	
Education opportunity	PvC	–	–	23.57	34.29	42.14	+
	PsC	23.93	63.57	12.5	–	-	
Social relationship	PvC	3.93	78.57	12.86	–	4.64	-
	PsC	–	7.14	73.57	2.5	16.79	
Flood shelter	PvC	–	2.86	7.14	37.86	52.14	+
	PsC	4.64	85.71	4.65	–	5.00	

PvC, previous condition; PsC, present condition; +, improved in the present location; -, decreased in the present location. Field survey.

The displaced people reported their opinion concerning different forms of opportunities and other civic amenities; almost half of the respondents revealed that communication system was very bad in their previous dwelling places. Still, 67.5% of respondents stated that the communication system is good in the present location. Also, respondents shared that sanitation amenities, drinking water, healthcare services, education opportunities, and flood shelter were comparatively good in the present location. On the contrary, a majority of the respondents (78.57%) stated that their social network was relatively good in the previous location. Concerning this, a respondent narrated his/her opinion as follows:

*“We are a poor man living in an underdeveloped place, where we could not arrange a minimal level of family affairs every day. But we were relatively lived there unitedly and tried to share our grief with the relatives and neighbors. But climate change and its associated hazards kicked us different places. As a result, our social network has somehow reduced, and we feel lonely after being displaced from our previous position. As well, we are experiencing some problems while managing our lives” (Interviewee# 13).*

### Perception of displaced individuals on the climatic variability

This section illustrates the people’s perception on changing patterns of climate variables caused by climate change over the 12 years. Among the various climate variables, the study respondents shared their responses to the differences, where about half of the respondents believed that rainfall season slightly decreased, whereas 25.71% of the respondents argued that rainfall season increased to some extent. On the other side, approximately half of the respondents (45%) reported that rain intensity increased rapidly (Table 3).

**TABLE 3:** People perception of climate change in the last 12 years.

Climatic variables	Respondent’s responses on changing patterns					
	Rapidly increased (%)	Slightly increased (%)	No change (%)	Slightly decreased (%)	Rapidly decreased (%)	Don’t know (%)
Rainfall season	16.43	25.71	–	46.07	7.14	4.64
Rain intensity	45.00	32.86	2.86	18.93	–	–
Winter temperature	40.36	55.36	–	2.14	–	2.14
Summer temperature	64.29	33.21	–	–	2.50	–
Frequency of flood	36.07	63.93	–	–	–	–
Frequency of cyclone	9.29	56.07	30.36	–	–	4.29
Severity of Riverbank erosion	41.79	58.21	–	–	–	–
Heavy fog	27.50	48.93	20.00	–	–	3.57
Safe drinking water scarcity	27.14	40.00	20.36	5.36	–	7.14
Outbreak of diseases	17.50	72.86	9.64	–	–	–

Field survey.

**FIGURE 3:**
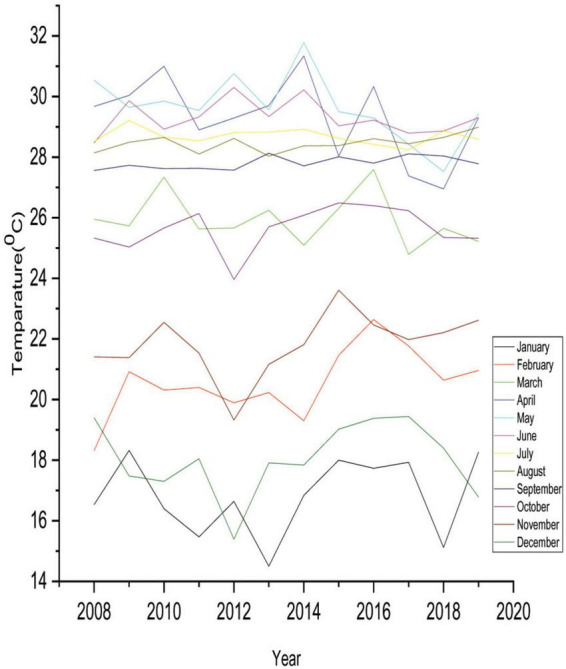
Monthly historical temperature pattern.

**FIGURE 4:**
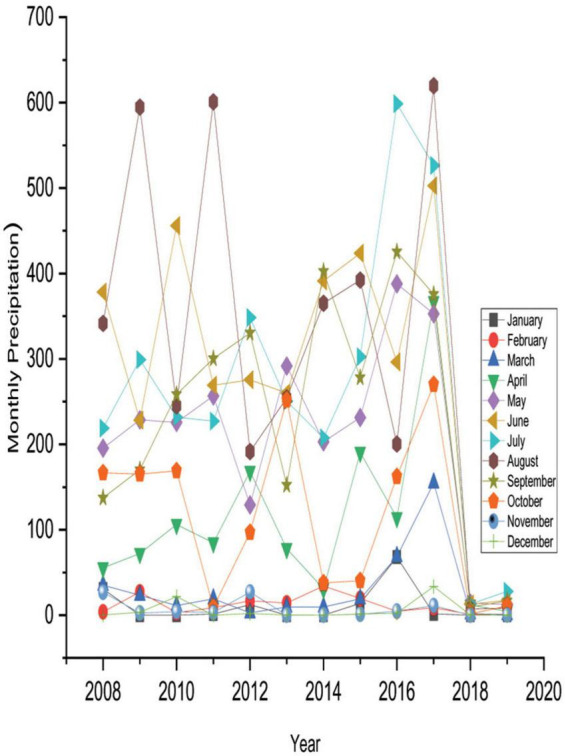
Monthly historical precipitation.

Table 3 also reveals that 40.36 and 55.36% of displaced people believed that temperature increased rapidly and slightly during winter. In addition, summer temperature also comparatively increased where the majority of the respondents (64.29%) observed that summer temperature rapidly increased. For this reason, 63.93, 56.07, and 58.21% of respondents believed that the frequency of flood disasters, cyclones, and intensity of riverbank erosion had relatively increased, respectively. On the contrary, 48.93% of IDPs unfolded that heavy fog slightly increased due to climate change, and 27.50% of respondents believed heavy fog rapidly increased. Safe drinking water in the disaster-prone areas had scarcity due to climate change and its extreme hazards. In this regard, 27.14% and 40% of IDPs conveyed that the scarcity of safe drinking water rapidly and slightly increased, respectively, in the study area. Therefore, the disease outbreak in the study areas enhanced massively due to the changing climate. The majority of respondents (72.86%) believed that numerous diseases relatively increased in the study zones because of climate change and its associated disasters. The analyzed results and the observed data from NASA Power were almost parallel to the perception of internally displaced people (Figures 3, 4).

### Perceived impact of climatic hazards on livelihood

The study revealed that changing temperature, rainfall pattern, and disaster have directly affect the IDPs’ livelihood over the last 12 years. Several livelihood capital problems, like human capital, social capital, financial capital, physical capital, and natural capital, were identified, and the impact intensity on livelihood capital is shown in Table 4, according to the IDPs’ perception. In the case of human capital issues of IDPs due to climate change, people faced food uncertainty and malnutrition, health issues, unemployment, education disruption, and knowledge and skill issues. Therefore, the study found that almost 44.6%, 30.4%, and 25% of the respondents were confronted with large, medium, and slight food and malnutrition problems, respectively, in the previous location. Conversely, 13.9% of respondents did not face any difficulties; 31.8, 39.6, and 14.6% of respondents encountered slight, medium, and large impacts of food uncertainty and malnutrition, respectively, in the present location. The people were comparatively more vulnerable in the previous location than in the present place in the case of health issues, where 7.5% of the respondents did not face any health problems in their earlier setting and 23.9% of the respondents did not confront any health problems in the present site. Furthermore, 31.8% of the respondents found no unemployment in their previous location, whereas only 9.6% of respondents claimed no unemployment in the present location. The education sector is relatively much better for the IDPs in the current location than in the previous place. Concerning knowledge and skill, the IDPs did not face extensive problems in their previous and present locations.

**TABLE 4:** Perception of displaced people on the climate change impact on livelihood in the prior and present locations.

Types of assets	Impacts	Description	Previous location (%)				Present location (%)			
			N	S	M	L	N	S	M	L
Human capital	Food uncertainty and malnutrition	Enlarged due to low production and income	–	25.0	30.4	44.6	13.9	31.8	39.6	14.6
	Disease/health condition	Due to household food uncertainty and inadequate access to health amenities, the vulnerable dwellers are susceptible to ailment and possess poor health.	7.5	17.1	34.6	40.7	23.9	36.1	31.4	8.6
	Unemployment	Reduced employment opportunities	31.8	38.9	17.1	12.1	9.6	20.0	46.1	24.3
	Education	Education facilities disrupts and damages	–	27.5	40.0	32.5	23.9	48.9	23.6	3.6
	Knowledge and skill	Disrupts of knowledge and skills of family members	40.7	31.4	22.1	5.7	12.1	31.1	12.9	8.2
Social capital	Social relationship	Deteriorating social relationship among the family members and beyond	73.6	15.0	11.4	–	10.0	27.5	24.3	38.2
	Organizational involvement	Complications of household members participation in the organizations	12.1	52.5	24.3	11.1	81.4	18.6	–	–
	Medical facilities	Problems to access to health services of the household’s members	–	11.4	35.0	30.0	22.1	38.6	23.6	15.7
Financial capital	Loan facilities	Access to formal and informal sources of loan decreased	15.7	20.0	36.1	28.2	35.0	40.0	20.0	5.0
	Occupation and income	Barrier to get occupation and income sources	23.9	18.6	35.0	22.5	18.2	34.6	36.8	10.4
	Savings	The ability to save has decreased due to low income	4.6	29.3	41.4	24.6	27.5	36.4	27.9	8.2
	Crops	Crop’s loss	–	4.3	28.2	67.6	40.0	34.6	25.4	–
Physical capital	Housing	Damage and destroy housing	4.6	9.3	40.4	25.7	63.6	23.9	12.5	–
	Sanitation facilitation	Worsened sanitation facilities	–	12.1	42.5	45.4	36.8	32.5	30.7	–
	Agricultural assets	Loss of household’s agricultural assets	27.5	31.8	37.5	3.2	75.7	24.3	–	–
	Non-agricultural equipment’s	Loss of household’s non-agricultural assets	4.3	35.0	39.6	21.1	78.2	21.8	–	–
	Electricity (Solar/DB)	Deteriorated energy services	20.4	31.8	40.0	7.9	75.7	16.8	7.5	–
Natural capital	Land	Loss of land	63.9	16.8	11.4	7.9	95.4	4.6	–	–
	Drinking water	Complications regarding the availability of safe drinking water	6.1	27.9	40.0	26.1	38.6	42.5	18.9	–
	Livestock	Loss of livestock and paucity of fodder and poor animal health	49.6	20.7	15.0	14.6	81.8	14.6	3.6	–
	Fisheries	Shortage of fish in pond	70.7	15.0	7.9	6.4	95.4	4.6	–	–
	Social forestry	Loss of homestead trees	63.9	20.4	9.6	6.1	92.5	7.5	–	–

Types of impacts: N, No.; S, slightly, M, medium; L, large. Field survey.

On the contrary, IDPs were confronted with huge problems regarding social capital, like deteriorating social relationships, complicated organizational involvement, and problems accessing medical facilities. Thus, most IDPs conveyed that they did not face social relationship problems in their previous location. By contrast, only 10% of IDPs did not face social relationship problems in the existing site. Regarding the medical facilities, the IDPs believed that they are getting satisfactory facilities in the present location than in the ancestral place. On the other hand, the IDPs faced enormous complications triggered by climate change and its associated hazards concerning financial capital, such as access to formal and informal sources of loan decreased, a barrier to get occupation and income sources, disruption in savings, and crop loss. Table 4 also shows that loan facilities, occupation, and income were less impacted in the current location than in the prior place. In addition, crop loss is also reduced in the current location than in their ancestral place. In the case of physical capital, damaged and destroyed housing, worsened sanitation, and loss of agricultural and non-agricultural assets were the key impacts on IDPs in their present and previous locations due to climate change and its associated hazards. In addition, Table 4 reveals that housing and sanitation damage and destruction are comparatively reduced in the current location than in the former place. On the contrary, 75.7 and 78.2% of the respondents reported that no agricultural and non-agricultural assets were lost in the existing location. By contrast, only 27.5 and 4.3% of respondents claimed no agricultural and non-agricultural assets were lost in the former location. Natural capital was also impacted due to climate change and its associated hazards. The loss of land, disruption in collecting pure drinking water, livestock loss, and so on were the pivotal impact on the IDPs in their previous and present locations.

### Adaptation strategies by the respondents to livelihood resilience

As the climate change impacts on the displaced riverine char dwellers are enormous, the vulnerable inhabitants usually carry out various adaptation strategies to livelihood resilience. Table 5 illustrates the adaptation strategies of IDPs to livelihood resilience.

**TABLE 5:** Adaptation strategies of IDPs in the context of livelihood resilience.

Adaptive measure	Responses (%)	Household income category (%)			Comments
		Low	Medium	High	
Reduce expenditure	92.50	xxx	xxx	xxx	PA/ILA
Reduce food consumption and storage of food	77.50	xxx	xxx	xxx	PA/ILA
Begging	24.29	xxx	xx	–	ILA
Housemaid servant	27.50	xxx	xxx	–	ILA
Work as day labor (farm related)	45.36	xxx	xxx	xx	ILA/PA
Goat rearing	70.36	xx	xxx	xxx	ILA/PA
Chickens and duck rearing	96.79	xxx	xxx	xxx	ILA/PA
Street hawker (tea, vegetables seller, etc.)	13.23	xxx	xxx	x	ILA
Off-farm work (Van, rickshaw, *nachimon*, *korimon* and tempo, driver	63.21	xxx	xxx	xx	ILA
Take treatment (Govt. hospital, Quack doctor)	100.00	xxx	xxx	xxx	ILA/PA
Use mosquito net to prevent vector-borne diseases	96.07	xxx	xxx	xxx	ILA/PA
Taking Borga	36.43	xx	xx	xxx	PA/ILA
Housing elements amenities (GOs and NGOs)	13.57	xxx	xxx	xxx	PA
Taking loan (NGOs, moneylender, relatives and neighbor)	98.93	xxx	xxx	xxx	PA/ILA
Schooling of children (Govt. primary school)	63.57	xxx	xxx	xxx	PA/ILA
Use community sanitation	27.50	xxx	xx	–	PA/ILA
Collect drinking water (Road side public tap, Tub well located in street/slums)	66.07	xxx	xxx	x	ILA
Take shelter (Khas land)	40.00	xxx	xxx	xx	ILA
Housing (rent and/or relatives homestead)	60.00	xxx	xxx	xxx	ILA

xxx, high popular; xx, medium popular; x, low popular; ILA, individual-level adaptation based on experience and knowledge; PA, planned adaptation (supported by GOs and NGOs).

Source: field survey; multiple responses have been considered.

Table 5 reveals that 92.50 and 77.50% of the respondents reduced expenditure and reduced food consumption and storage of food, respectively. These are the most popular strategies for sustainable adaptation, and all categories of households followed this to counter climate change and its associated hazards. In addition, a majority of IDPs engaged in several occupations to adapt to the overwhelming condition, such as begging (24.29%), housemaid servant (27.50%), work as day labor (45.36%), street hawker (13.23%), and off-farm worker (63.21%). Mostly, these occupations are prevalent in low and medium households, and the FGDs revealed that IDPs try to find this work every day. On the contrary, most households are rearing goat (70.36%) and chicken and duck (96.79) after the displacement from their ancestral place. FGDs unfolded that rearing goat, chicken, and duck is a rapid source of financial solvency, and it is also a major source of rich food.

All the respondents usually visit government hospitals and quack doctors to treat illness as they did not have such healthcare facilities in their earlier location. Moreover, 96.07% of respondents use a mosquito net to preclude vector-borne ailments since they are not living in hygienic places, and their houses are fragile. In addition, 40% and 60% of the respondents have made their housing to live in Khas land (Khas land is a state-owned fallow land where nobody has property rights.), rent, and relative homestead. On the other hand, 66.07% of IDPs are collecting drinking water from road side public taps and tube wells located in their street/slum, and it is very popular between the low- and middle-income households. Moreover, FGDs revealed that children of IDPs are comparatively getting the opportunity for education in the present location than in the previous location, and almost 63.57% of the children attend the government primary school.

Furthermore, approximately all the IDPs revealed that they took a loan from NGOs, money lenders, relatives, and neighbors. This strategy was followed by almost all households after the displacement for the financial affluence to cope with the adverse situation. Also, some of the households (13.57%) received housing elements amenities from the GOs and NGOs. Mainly, most IDPs do not have cultivable land, so 34.43% of the respondents claimed that they took Borga (sharecropping) to cultivate different crops. Therefore, it can be said that the IDPs took various adaptation measures to cope with climate change and its associated numerous complications after being displaced from the ancestral place.

### Factors hindering the livelihood adaptation strategies

**FIGURE 5:**
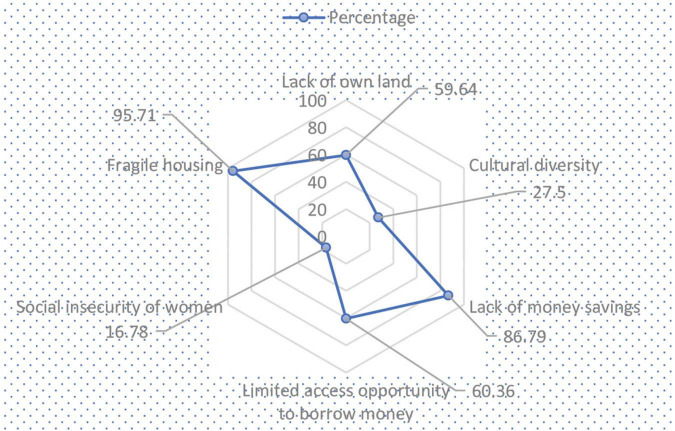
Radar chart on components of adaptation obstacles to livelihood resilience.

Since the IDPs formed several adaptation strategies to prevent the overwhelming situation, they also identified various barriers against the adaptation strategies. Figure 5 delineates the components of adaptation obstacles to the livelihood resilience of the displaced people.

Figure 5 shows that most respondents (59.64%) had not owned a land. This is why they faced enormous complications to adapt to the adverse situation after the displacement. A respondent talked concerning this as follows:

*“I am a poor man. I was living in the riverine char and working as day labor. I did not have cultivate land except my homestead. We were happy with my family but one day our happiness had ended because of riverbank erosion. We lost our homestead as well as household assets. Then, we had looked for a place to live but did not find a place near our home. After that, we came here to live but we are facing some problems including homestead land, which has disrupted our lives massively”(Interviewee# 25).*

Overall, 95.71 and 86.79% of the respondents believed that fragile housing and lack of saving money, respectively, were the pivotal barriers to adaptation in the face of crisis after the displacement. The FGDs revealed that the displaced people were not financially strong enough to manage life and livelihood issues. For this reason, most of the IDPs could not make sustainable housing due to financial problems. However, approximately 27.5% and 16.78% of the respondents reported that women’s cultural diversity and social insecurity were also significant barriers to adapting to the displacement’s adverse circumstances. On the contrary, 60.36% of the IDPs had limited opportunity to borrow money, which was also one of the obstacles. Regarding this matter, a respondent stated his/her opinion as follows:

*“We are a homeless individual living in the demesne (Khas land). In fact, we don’t have liquid money to cope with the complications. Still, at least we were able to manage some money from different sources like Mahajan, local NGOs, relatives and so on at our earlier location. But here, we have not received financial help as a loan from anywhere. We communicated some of the sources, but we could not meet up their requirement to get money. Although some of the individuals (Mahajan) agreed to provide us with some money, the interest rate was very high, as we are poor how we can repay the money at high interest. For this reason, we are confronting endure a lot of barriers to tackle the dynamic problems of life and livelihood” (Interviewee# 44).*

## Discussion

There is much evidence showing that climate change has a severe impact on displaced people in Bangladesh, along with the increasing severity and occurrence of catastrophes (IPCC, 2014; Alam et al., 2017). Field observation in Saghata and Fulchari also portrayed that owing to scarcity of social kinship and possessions, there is limited earning opportunity, which makes displaced people most susceptible. Although some of them can manage their earnings, their incomes are insufficient to support a family, which impacts their quality of life and health issues. Livelihood challenges are faced by the char dwellers due to a lack of education, less work opportunity, and low earnings, which is consistent with the outcomes of a study directed by Ahmed and Atiqul Haq (2019) and Haque et al. (2020).

The study exposed that six drivers induced massive displacement in the study area. The drivers and factors of displacement are considerably influenced promptly by climate change and its associated hazards. This study revealed that the main reasons for escalating displacement in Saghata and Fulchari upazilas are frequent flood disasters, riverbank erosion, and crop loss. Riverine char regions of Bangladesh are vulnerable to various calamities, such as flood disasters, riverbank erosion, and drought. For this reason, people are confronted with huge losses regarding lives and livelihood every year. In this case, Zaman (1989) and Alam et al. (2020) also identified that the internal migration is rising in riverine char regions of Bangladesh owing to implications of these sorts of catastrophes. Conversely, Barua et al. (2017) and Shi et al. (2019) examined that financial difficulties are strongly linked to internal migration in developing nations. According to this study, internal migration was found to be increasing due to the loss of livelihood and harm to agriculture. Also, in this study, social services and livelihood opportunities available in the former and current places were account for after their displacement. Considering societal amenities, Islam and Hasan (2016) and Kabir et al. (2018) explained that defenseless people relocate to developed areas where communication system, drinking water accessibility, and flood living quarters were available. However, the displaced people of char areas need time to adjust to society and form positive relationships. Conflict arises among IDPs in some circumstances due to a lack of shared resources, and nearby settled people refuse to grant access to their lands. Apart from all of these considerations, migrants usually evaluate their family situation and road communication conditions while determining their migration destination and period (Martin et al., 2014).

According to the findings, the IDPs detected changes in growing or reducing trends for various climatic factors at their current locale compared to their former places. Likewise, Hossain et al. (2021) indicated that temperature, precipitation, and calamities are shifting an intense stage. Nishat and Mukherjee (2013) mentioned that in current years, Bangladesh has seen an increasing trend of escalating temperatures and various sorts of calamities and a shifting form of yearly precipitation. Rahman and Lateh (2017) noticed that the average temperature of Bangladesh is increasing at a rate of 0.20 °C per decade, indicating an upward trend in temperature, which is consistent with displaced people’s perceptions of the rising temperatures. Likewise, to highlight the possibility of natural catastrophes in Bangladesh, District Disaster Management Committee (2014) predicted that floods and cyclones would increase because of the increasing tendency of sea surface temperature and rainfall.

In the northern zone, particularly in char areas of Bangladesh, approximately all households confronted life and livelihood complications caused by climate change and its associated hazards. Since they are directly and indirectly dependent on agriculture, this sector is impacted enormously triggered by extreme calamities regularly. Islam (2018) and Hossain et al. (2020a) stated that flood disasters and riverbank erosion are the pivotal reasons for encountering life and livelihood difficulties in the char areas. The present study found that the IDPs addressed several short- or long-term challenges concerning life and livelihood. These rising livelihood complications are due to changing climate such as increased rainfall season and rain intensity, frequent flood disaster and riverbank erosion, and crop loss. Alam et al. (2020) delineated that several impacts like food shortage, unemployment, and disruption of education due to climate change are found in the char areas. The IDPs have also reported the same problems they faced in the present and previous locations. In addition, they identified that food security and education facilities at the present locations were higher than those in their previous regions. On the contrary, many respondents unfolded that they suffered from several health issues, such as vector-borne and waterborne diseases, which is coherent with other studies (Pyle et al., 2009; Hossain et al., 2021). Furthermore, the IDPs believed that they encountered numerous diseases in both locations, which impedes their sustainable way of life. Furthermore, the displaced people addressed that after being displaced to a new place, there has been some deterioration in social relations between family members as well as members of the new society, which is consistent with the findings of the research directed by Hynie (2018) and Nikuze et al. (2019). In addition, Hossain et al. (2020a) discussed that the IDPs seemed to be helpless and hopeless after being displaced. A lack of intact social relationships made them more fragile and vulnerable. In addition, the study respondents reported that they faced several types of social, financial, physical, and natural capital problems due to the changing climate and its associated hazards both at the new and previous locations, which is similar to research conducted by other researchers (Alam et al., 2017; Islam and Shamsuddoha, 2017).

Adaptations at different levels using both physical and environmental strategies are required to maintain a viable way of life in the face of climate change consequences (Munroe et al., 2012). The IDPs have adopted several adaptation strategies in terms of their household income capacities to cope with the adverse situations. Similarly, Barua et al. (2017) and Jha et al. (2018) disclosed that different adaptation measures followed by the displaced people formulated based on the individuals’ level or planned adaptation supported by GOs and NGOs could be worthwhile to ensure the sustainable livelihood against the climate change penalties. In addition, Barua et al. (2017) stated that these adaptation practices make an effort to diminish the displacement risk for the households in future caused by climate change and its amalgamated hazards. Also suggested taking rearing livestock, petty business, visiting doctors, off-farm working, employing traditional practices, and consulting relatives, friends, and neighbors to cope with the shifting pictures of climate change effects on life and livelihood. Following from the significance of adaptation in securing sustainable livelihood in the face of climate change, Brouwer et al. (2007) and Hossain et al. (2020a) emphasized the inevitability of executing appropriate adaptation mechanisms through long-term measures in less developed nations like Bangladesh. Women, youths, and the elderly are the groups that demand special concentration to properly carry out the acclimatization programs, where school-based learning would emphasize climate change and livelihood adjustment (Kabir M. I. et al., 2016; Luetz, 2018).

On the other hand, identifying the obstacles to adaptation in the face of climate change is essential for designing effective livelihood coping mechanisms (Alemayehu and Bewket, 2017). The fragile financial status among the various shapes of obstacles is the most underlined factor, which diminishes the adjustment competencies (Moser and Ekstrom, 2010; Hossain et al., 2020a). Likewise, the monetary crisis is one of the pivotal barriers for the displaced people in Saghata and Fulchari regions. A majority of respondents are engaging as a rickshaw puller, day labor, beggar, bricklayer, and street hawker, and so on. In addition, respondents also reported some of the barriers, such as lack of land and fragile housing, to cope with livelihood issues, as found in the research conducted by Nikuze et al. (2019).

This study findings have significant policy implications for countries like Bangladesh. The outcomes of this study have immediate implications for countrywide policy priorities such as poverty reduction, ultra-poor improvement, island livelihood projects, and distinct assistance for socially excluded people. Acute poverty in disaster-prone regions like char is one of the key points in the poverty alleviation strategy of the Bangladesh government (International Monetary Fund, 2005). The government recognizes that the displaced people in disaster-prone areas are rigorously underprivileged based on land rights, paucity of right to use to formal finance, and other fundamental facilities (Paul and Islam, 2015). The Sustainable Development Goals (SDG) in Bangladesh have an explicit purpose of lessening extreme poverty in the countryside areas. Therefore, climate change and disasters, including socioeconomic vulnerabilities and other risks faced by island dwellers and/or displaced people, must be mapped to improve their ability to recover and adapt to threats. In addition, potential disaster resilience guides should be formulated to categorize an institute systems, measures, and functioning circumstances in the aftermath of calamities. The study outcomes noticeably display that owing to their little human assets, economic vulnerabilities, and so on, the respondents had no choice but to relocate to the mainland or surrounding towns.

## Conclusion

Each year, a huge number of individuals internally displace in Bangladesh, where riverine char regions are highly prone to extreme flooding, riverbank erosion, etc. Among the overall population of the disaster-prone areas in Bangladesh, char dwellers are one of the most vulnerable people to natural disasters, including changes in other climatic factors, which induce them massively to be displaced. For this reason, the displaced char inhabitants confront enormous complications with regard to lives and livelihood without discriminating, which make them even more vulnerable. This study aimed to examine the impact on the lives and livelihoods of IDPs caused by climate change, including local adaptation mechanisms. It is revealed that the char dwellers are displaced to a new place from their ancestral locations due to climate-induced disasters like floods, riverbank erosion, and their consequences. The relocated people reported facing huge impediments such as housing and sanitation problems, food uncertainty, health problems, and various social issues after being displaced in a new place. Even though they formulated copious adaptation strategies like reducing expenditure and food consumption, begging, livestock rearing, and taking shelter in demesne land to eliminate their livelihood issues and to sustain lives. The livelihood resilience strategies of displaced people were interrupted owing to numerous obstacles, such as paucity of money, lack of own land, fragile housing, and social insecurity. Furthermore, the adaptation strategies of displaced people vary based on a household’s income capacity since most families’ financial condition is not good enough.

However, GOs and NGOs, including all people working for vulnerable people, should use a significant development strategy to ensure a viable adaptation approach is established and various forms of obstructions for the IDPs are evaded. On the other hand, future studies could be conducted with numerous displaced people in many disaster-prone zones since this study was carried out in only two upazilas of Gaibandha district. Also, a longitudinal study could also be carried out to understand the thorough scenarios of the climate-induced displacement of people.

## Data availability statement

The original contributions presented in this study are included in the article/supplementary material, further inquiries can be directed to the corresponding author.

## Ethics statement

Studies involving human participants were reviewed and approved by the Behavioral and Social Sciences Ethical Review Committee (BSSERC) of Hohai University, Nanjing, China. Written informed consent from the patients/participants or patients/participants’ legal guardian/next of kin was not required to participate in this study in accordance with the national legislation and the institutional requirements.

## Author contributions

BH initiated the study. BH, MNIS, GS, and MSS collected the data and wrote the manuscript. BH, GS, and CA processed the data and performed statistical analysis. BH, MNIS, CA, ZS, and QY revised the manuscript. All authors read and approved the final manuscript.
